# Fluoride Alters Gene Expression via Histone H3K27 Acetylation in Ameloblast-like LS8 Cells

**DOI:** 10.3390/ijms25179600

**Published:** 2024-09-04

**Authors:** Shohei Yamashita, Motoki Okamoto, Melanie Mendonca, Natsumi Fujiwara, Eiko Kitamura, Chang-Sheng Sam Chang, Susanne Brueckner, Satoru Shindo, Nanako Kuriki, Marion A. Cooley, Navi Gill Dhillon, Toshihisa Kawai, John D. Bartlett, Eric T. Everett, Maiko Suzuki

**Affiliations:** 1 Department of Oral Science and Translational Research, College of Dental Medicine, Nova Southeastern University, Fort Lauderdale, FL 33314, USA; syamashi@nova.edu (S.Y.); mokamoto@nova.edu (M.O.); mm5650@mynsu.nova.edu (M.M.); sbrueckn@nova.edu (S.B.); sshindo1@nova.edu (S.S.); nokamoto@nova.edu (N.K.); tkawai@nova.edu (T.K.); 2 Biology I Halmos College of Arts and Sciences, Behavioral Neuroscience I College of Psychology, Nova Southeastern University, Fort Lauderdale, FL 33314, USA; 3 Department of Oral Health Care Management, Graduate School of Biomedical Sciences, Tokushima University, Kuramoto, Tokushima 770-8504, Japan; nfujiwara@tokushima-u.ac.jp; 4 Georgia Cancer Center, Augusta University, Augusta, GA 30912, USA; kitamue2@hotmail.com (E.K.);; 5 Department of Oral Biology and Diagnostic Sciences, The Dental College of Georgia, Augusta University, Augusta, GA 30912, USA; mcooley@augusta.edu; 6 Department of Biological Sciences, Halmos College of Arts and Sciences, Nova Southeastern University, Fort Lauderdale, FL 33314, USA; ngill@nova.edu; 7 Division of Biosciences, College of Dentistry, Ohio State University, Columbus, OH 43210, USA; bartlett.196@osu.edu; 8 Department of Biomedical Sciences, Adams School of Dentistry, The University of North Carolina at Chapel Hill, Chapel Hill, NC 27599, USA; eric_everett@unc.edu

**Keywords:** epigenetics, ChIP-Seq, histone modification, HAT, HDAC, ameloblasts, amelogenesis, dental fluorosis

## Abstract

Excessive fluoride ingestion during tooth development can cause dental fluorosis. Previously, we reported that fluoride activates histone acetyltransferase (HAT) to acetylate p53, promoting fluoride toxicity in mouse ameloblast-like LS8 cells. However, the roles of HAT and histone acetylation status in fluoride-mediated gene expression remain unidentified. Here, we demonstrate that fluoride-mediated histone modification causes gene expression alterations in LS8 cells. LS8 cells were treated with or without fluoride followed by ChIP-Seq analysis of H3K27ac. Genes were identified by differential H3K27ac peaks within ±1 kb from transcription start sites. The levels of mRNA of identified genes were assessed using rea-time PCR (qPCR). Fluoride increased H3K27ac peaks associated with *Bax*, *p21*, and *Mdm2* genes and upregulated their mRNA levels. Fluoride decreased H3K27ac peaks and *p53*, *Bad*, and *Bcl2* had suppressed transcription. HAT inhibitors (Anacardic acid or MG149) suppressed fluoride-induced mRNA of *p21* and *Mdm2*, while fluoride and the histone deacetylase (HDAC) inhibitor sodium butyrate increased *Bad* and *Bcl2* expression above that of fluoride treatment alone. To our knowledge, this is the first study that demonstrates epigenetic regulation via fluoride treatment via H3 acetylation. Further investigation is required to elucidate epigenetic mechanisms of fluoride toxicity in enamel development.

## 1. Introduction

Fluorine is a trace element found naturally in water, soil, plants, rocks, and air. Fluoride is effective in reducing dental caries, and fluoridation of public water supplies has been identified as the most practical and cost-effective method for preventing dental caries [1]. The U.S. Public Health Service (PHS) recommends public water fluoridation with an optimal fluoride concentration in drinking water of 0.7 ppm [1]. However, excessive systemic exposure to fluoride (over 1.5 ppm fluoride in drinking water) can cause dental and skeletal fluorosis [2]. There are several dental and skeletal fluorosis endemic regions in the world with parts of Asia and Africa affected by high fluoride concentrations (varying from 20 to 79.2 ppm F^−^) in the groundwater [3]. 

Dental fluorosis is caused by ingestion of greater than optimal concentrations of fluoride during enamel formation (amelogenesis), at around the ages of 8 years and younger [1]. Clinically, mild cases of dental fluorosis manifest as a white opaque enamel, caused by increased subsurface porosity [4]. Individuals with moderate dental fluorosis show yellow-to-light-brown staining in the areas of enamel damage. In very severe cases, the enamel is porous, poorly mineralized, stains brown, and contains relatively less minerals and more proteins than sound enamel [5,6]. Amelogenesis occurs in stages (e.g., the secretory stage and the maturation stage). During the maturation stage, ameloblasts, the cells responsible for enamel formation, are in direct contact with the acidic (pH < 6.0) mineralizing enamel matrix [7], suggesting that ameloblasts in the maturation stage are exposed to fluoride under low-pH conditions. The low-pH environment of the maturation stage facilitates the conversion of fluoride ions (F^−^) to hydrofluoric acid (HF) and promotes the entry of toxic HF into ameloblasts, which promotes fluoride-induced cell stress [8]. Compared to the neutral environment in the secretory stage (pH ~ 7.2), the low-pH environment of the maturation stage reduces the threshold dose required to induce fluoride-mediated cytotoxicity *in vivo*. In contrast, the *in vitro* cell culture environment maintains a neutral pH (~7.3), necessitating a higher fluoride dose to induce fluoride cytotoxic effects compared to the acidic maturation stage environment. Therefore, our in vitro experiments studied concentrations of 0 to 5 mM NaF (equivalent to approximately 0 to 43 ppm F^−^) [9,10,11].

Epigenetics is the study of heritable and stable changes in gene expression resulting from changes in a chromosome without alterations in the DNA sequence [12]. Epigenetic modifications include histone modification (acetylation and deacetylation), DNA methylation, and non-coding RNAs that activate multiple signaling pathways and regulate gene expression [13,14]. Histone acetylation is regulated by histone acetyltransferases (HATs) and histone deacetylases (HDACs) [15]. HATs add acetyl groups onto lysine residues within lysine-rich amino-terminal tails of histone proteins, resulting in activation of gene expression [16]. HAT inhibitors, such as anacardic acid (AA: inhibitor of CBP/p300 and PCAF) and MG149 (inhibitor of Tip60/KAT5), reduce acetylation levels of histones and suppress transcriptional activity [17,18]. We previously reported that fluoride activates HATs to increase p53 acetylation (p53ac), which promotes fluoride toxicity in mouse ameloblast-like LS8 cells [19]. HAT inhibitors (AA or MG149) suppress fluoride-mediated p53ac to inhibit apoptosis and DNA damage in LS8 cells [19]. In contrast, HDACs are enzymes that eliminate acetyl groups from 1-N-acetyl lysine amino acids on histones, restoring the histone to its basal state and silencing gene expression [20]. HDACs are grouped into four categories (class I, II, III, and IV). These classes differ in structure, localization, and cofactor requirements [21]. Class III HDACs, commonly known as sirtuins (SIRT1–7), cause hypoacetylation and control multiple cell signaling pathways. Unlike other classes of HDACs, which are zinc-dependent enzymes, the SIRT family consists of NAD^+^-dependent enzymes [22]. Previously, we have revealed that SIRT1 is involved in the adaptive response to fluoride-mediated cell stress [9]. SIRT1 overexpression in vitro suppresses fluoride-induced p53 acetylation to mitigate fluoride toxicity in LS8 cells [11]. Sodium butyrate (SB), one of the most investigated HDAC inhibitors (HDACis), is a short-chain fatty acid produced by gut microbiota. SB competitively binds to zinc sites in the catalytic region of HDACs and can influence cellular processes such as cellular proliferation, differentiation, and gene expression by regulating histone deacetylation [23]. For example, SB increases histone acetylation levels at gene promoters to increase the expression of anti-inflammatory genes by inducing the expression of genes downstream of the IL10/STAT3 anti-inflammatory pathway to mitigate microglia-mediated neuroinflammation [24]. Recent studies have demonstrated that SB inhibits class III HDACs to promote cancer cell apoptosis [25].

This study aims to investigate the roles of HATs and HDACs in fluoride-mediated histone acetylation and gene expression in LS8 cells. For the first time, we demonstrated that fluoride regulates epigenetic processes by modifying histone acetylation, which alters gene expression in ameloblast-like cells.

## 2. Results

### 2.1. Detection of Gene Locations with Differential H3K27ac Occupancy Following Fluoride Treatment

**Figure 1:**
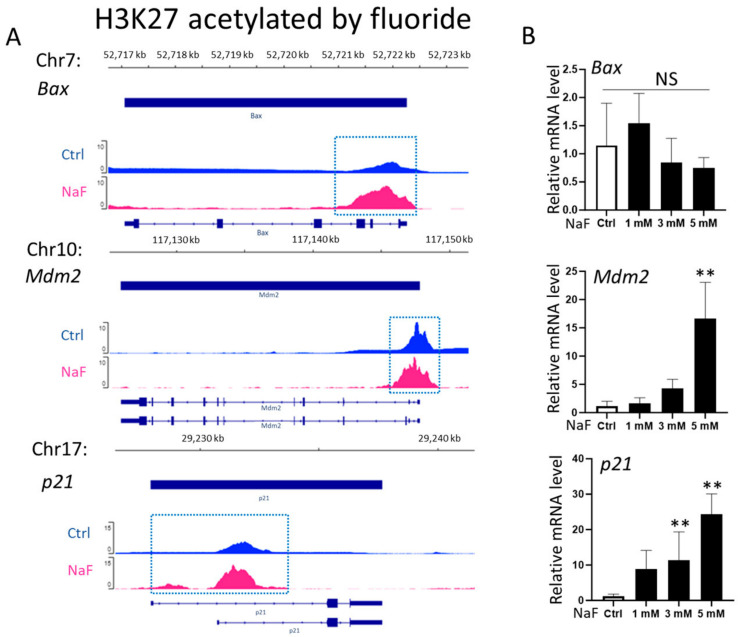
Analysis of gene expressions associated with acetylation of H3K27 via fluoride in LS8 cells. LS8 cells were treated with sodium fluoride (NaF) (0 to 5 mM) for 24 h. (**A**) Integrative Genomics Viewer (IGV) images show H3K27ac patterns of *Bax*, *Mdm2*, and *p21* from ChIP-Seq. *Y*-axis denotes ChIP signal amplitude, while *x*-axis indicates genome positions. The blue dotted box shows the increase in the peak area in the promoter regions induced with 5 mM NaF treatment. (**B**) The mRNA levels of *Bax*, *p21*, and *Mdm2* were analyzed using real-time PCR (qPCR) (N = 4/group). Data are presented as means ± SD. ** *p <* 0.01, NS; no significant differences.

**Figure 2:**
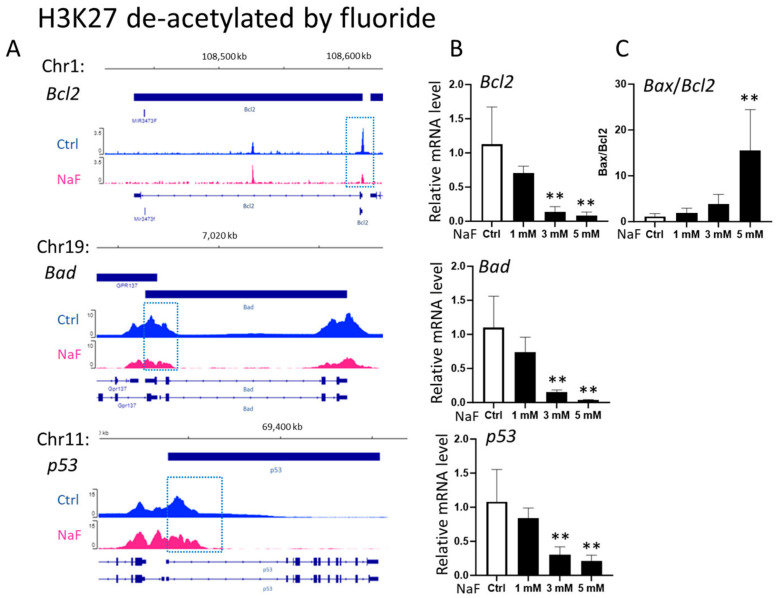
Analysis of gene expressions associated with deacetylation of H3K27 via fluoride in LS8 cells. LS8 cells were treated with NaF (0 to 5 mM) for 24 h. (**A**) Integrative Genomics Viewer (IGV) images show H3K27ac patterns of *Bcl2*, *Bad*, and *p53* from ChIP-Seq. *Y*-axis denotes ChIP signal amplitude, while *x*-axis indicates genome positions. The blue dotted box shows the reduced peak area in the promoter regions induced via 5 mM NaF treatment. (**B**) The mRNA levels of *Bcl2*, *Bad*, and *p53,* and (**C**) the *Bax*/*Bcl2* mRNA ratio were analyzed using qPCR (N = 4/group). Data are presented as means ± SD. ** *p <* 0.01.

LS8 cells were treated with 0 to 5 mM sodium fluoride (NaF) for 24 h. ChIP-Seq was performed to identify the genome-wide H3K27 acetylation status (H3K27ac) within ± 1 kb of transcription start sites (TSS). The promoter regions of *Bax*, *Mdm2*, and *p21* had increased levels of H3K27ac peak areas after exposure to 5 mM NaF (pink) compared to untreated controls (blue) (Figure 1A). Conversely, the H3K27ac peak areas in the promoters of *Bcl2*, *p53*, and *Bad* were reduced by NaF (pink) compared to controls (blue) (Figure 2A). Table 1 shows gene locations associated with H3K27ac peaks that were increased (*Bax*, *Mdm2* and *p21*) or decreased (*Bcl2*, *p53* and *Bad*) due to fluoride treatment.

### 2.2. Comparison of Gene Expressions and H3K27ac Occupancy Following Fluoride Treatment

Fluoride effects on transcript levels of *Bax*, *Mdm2*, *p21*, *Bcl2*, *Bad*, and *p53* were analyzed via quantitative real-time PCR (qPCR). LS8 cells were treated with or without 5 mM NaF for 24 h. NaF treatment significantly increased transcript levels of *Mdm2* and *p21* dose dependently compared to untreated controls (*p <* 0.01) (Figure 1B)*,* which was associated with elevated levels of H3K27ac in their promoter regions (Figure 1A). However, *Bax* mRNA expression was not changed due to NaF at any doses (Figure 1B). NaF treatment significantly decreased mRNA expression of *Bcl2*, *p53*, and *Bad* dose dependently compared to untreated controls (*p <* 0.01) (Figure 2B)*,* which was associated with decreased H3K27ac levels in their promoter regions (Figure 2A). Except for *Bax*, the H3K27ac status in promoter regions were concordant with mRNA expressions, suggesting that fluoride alters gene expressions of *Mdm2, p21*, *Bcl2*, *p53*, and *Bad* via H3K27 acetylation. The mRNA ratio of *Bax*/*Bcl2* reflects the balance between pro-apoptotic (Bax) and anti-apoptotic (Bcl2) proteins [26]. Although *Bax* mRNA levels were not changed via NaF treatment (Figure 1B), the mRNA ratio of *Bax/Bcl2* was significantly increased with NaF treatment (*p <* 0.01) (Figure 2C). This suggests that fluoride-induced apoptosis is caused by the suppression of *Bcl2* expression, which is associated with the reduction in H3K27ac in its promoter region (Figure 2A,B).

### 2.3. Fluoride-Induced p21 and Mdm2 mRNA Levels Are Suppressed by HAT Inhibitors (AA, MG149)

**Figure 3:**
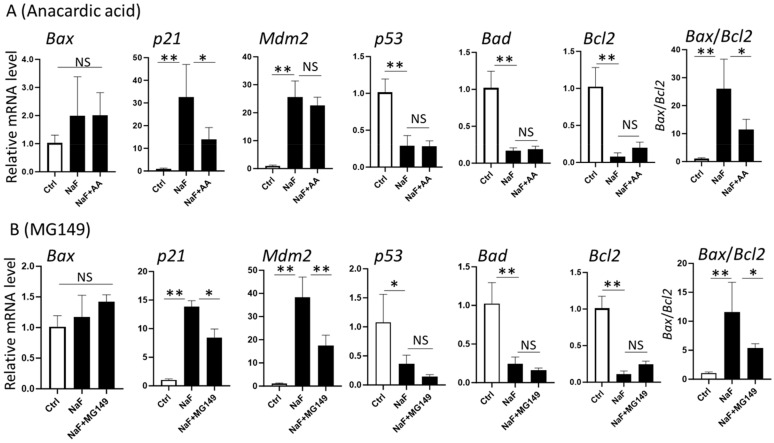
Effects of histone acetyltransferase (HAT) inhibitors (Anacardic acid; AA and MG149) on gene expressions associated with histone H3K27 acetylation via fluoride. LS8 cells were treated with 50 μM AA or 50 μM MG149 for 1 h prior to 5 mM NaF treatment for 24 h. mRNA levels were evaluated via qPCR (N = 5/group for AA, N = 4/group for MG149). (**A**) Addition of AA to NaF significantly decreased mRNA of *p21* and the *Bax*/*Bcl2* mRNA ratio compared to NaF treatment alone, but other genes were not changed with the addition of AA. (**B**) Addition of MG149 to NaF significantly decreased mRNA of *p21* and *Mdm2* and the *Bax*/*Bcl2* mRNA ratio compared to NaF treatment alone, but other genes were not changed by MG149 addition. Control (Ctrl) and NaF included vehicles (DMSO 0.1% for AA and 0.04% for MG149). Data are presented as means ± SD. * *p <* 0.05, ** *p <* 0.01, NS; no significant differences.

We reported previously that fluoride increases histone acetyltransferase (HAT) activity of CBP/p300 and Tip60/KAT5 in LS8 cells [19]. Here, we examined the roles of HAT in fluoride-induced gene expression alterations, which are associated with H3K27ac status, by using HAT inhibitors: anacardic acid (AA: inhibitor of CBP/p300 and PCAF) and MG149 (inhibitor of Tip60/KAT5). AA and MG149 can suppress the acetylation of histone and non-histone proteins. LS8 cells were treated with 5 mM NaF with or without AA (50 μM) or MG149 (50 μM) for 24 h. The addition of AA significantly suppressed *p21* mRNA expression compared to NaF alone (*p <* 0.05) (Figure 3A), but AA did not change *Mdm2* mRNA expression compared to NaF alone. The addition of MG149 significantly decreased both mRNA expressions of *p21* (*p <* 0.05) and *Mdm2* (*p <* 0.01) compared to NaF alone (Figure 3B). These results suggest that Tip60/KAT5 is involved in fluoride-mediated histone acetylation and mRNA expression in both *p21* and *Mdm2* genes (Figure 3B), whereas CBP/p300 and PCAF are involved in *p21* gene expression but not in *Mdm2* expression (Figure 3A). NaF suppressed *p53*, *Bcl2*, and *Bad* mRNA expression, which is associated with the downregulation of H3K27 acetylation in these genes (Figure 2). Gene expression of *p53*, *Bcl2*, and *Bad* was not changed by the addition of AA (Figure 3A) or MG149 (Figure 3B) compared to treatment with NaF alone, indicating that fluoride-mediated suppression of these genes is independent of HATs. NaF treatment (5 mM) significantly increased the ratio of *Bax/Bcl2* compared to the control (Figure 2C). This increase was suppressed by AA or MG149 (*p <* 0.05) (Figure 3), indicating that the inhibition of HATs can mitigate fluoride-mediated apoptosis.

### 2.4. Fluoride Suppresses the Activity of Class I and II HDACs

**Figure 4:**
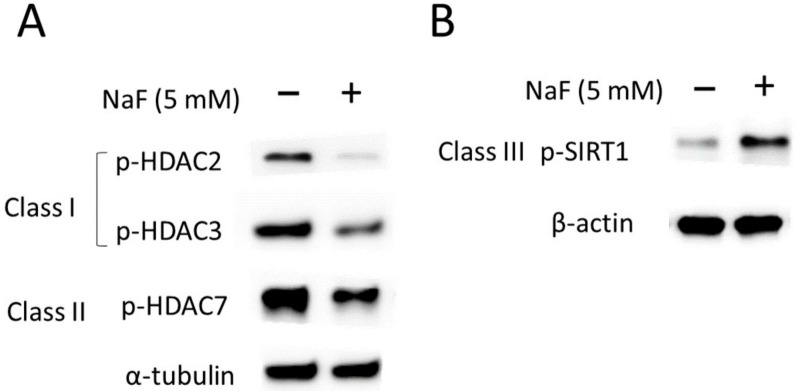
The effect of fluoride on class I, II, and III histone deacetylases (HDACs) in LS8 cells. LS8 cells were treated with 5 mM NaF for 24 h. The protein levels of phosphorylated HDACs, p-HDAC2 (Ser394) (62 kDa), p-HDAC3 (Ser424) (49 kDa), p-HDAC7 (Ser155) (124 kDa), and p-SIRT1 (Ser47) (82 kDa) were detected via western blot (WB). (**A**) Fluoride treatment attenuated the protein levels of class I HDACs (p-HDAC2 and p-HDAC3) and class II HDAC, p-HDAC7. (**B**) Class III HDAC, p-SIRT1, expression was increased with fluoride treatment. α-Tubulin (52 kDa) and β-actin (44 kDa) were used as loading controls. Representative images are shown. Quantification and statistical analyses of relative protein levels are shown in Supplementary Figure S1A.

We have previously reported that fluoride activates a class III HDAC, SIRT1, as an adaptive response to fluoride-mediated cell stress [9]. However, how fluoride alters the activity of class I and II HDACs is not clear. To address this, we examined the effects of fluoride on HDACs. Phosphorylated-HDACs (p-HDAC or p-SIRT1) are the active forms of these deacetylases contributing to deacetylase activity, which may repress transcription and protein interactions [27]. LS8 cells were treated with 5 mM NaF for 24 h and phosphorylated HDACs (p-HDAC or p-SIRT1) were detected via Western blot. NaF significantly decreased the levels of active HDACs, including class I HDACs (p-HDAC2 and p-HDAC3) and class II HDAC (p-HDAC7) compared to untreated controls (*p <* 0.01) (Figure 4A and Figure S1A). In contrast, fluoride significantly increased active p-SIRT1 levels compared to controls (*p <* 0.01) (Figure 4B and Figure S1A). These results suggest that fluoride-mediated suppression of class I and II HDAC activity contributes to increased histone acetylation, while fluoride-mediated SIRT1 activation leads to deacetylation of histones. 

### 2.5. Sodium Butyrate Suppresses the Activity of Class I, II, and III HDACs and Increases Histone Acetylation

**Figure 5:**
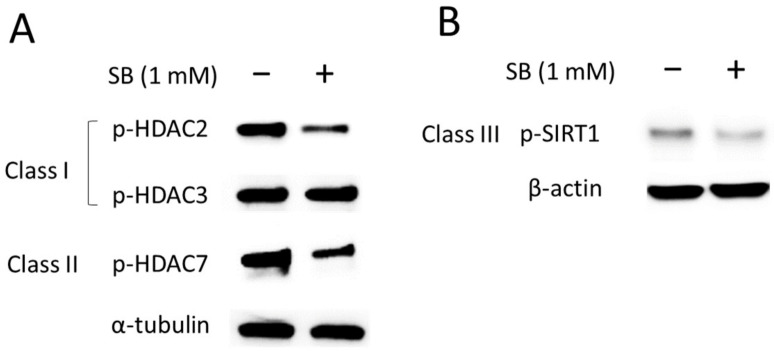
The effect of sodium butyrate (SB) on class I, II, and III HDACs in LS8 cells. LS8 cells were treated with 5 mM NaF for 24 h. The protein levels of phosphorylated HDACs, p-HDAC2 (Ser394) (62 kDa), p-HDAC3 (Ser424) (49 kDa), p-HDAC7 (Ser155) (124 kDa), and p-SIRT1 (Ser47) (82 kDa) were detected via WB. SB attenuated the protein levels of p-HDAC2, p-HDAC7 (**A**), and p-SIRT1 (**B**). SB did not change p-HDAC3 (**A**). α-Tubulin (52 kDa) and β-actin (44 kDa) were used as loading controls. Representative images are shown. Quantification and statistical analyses of relative protein levels are shown in Supplementary Figure S1B.

**Figure 6:**
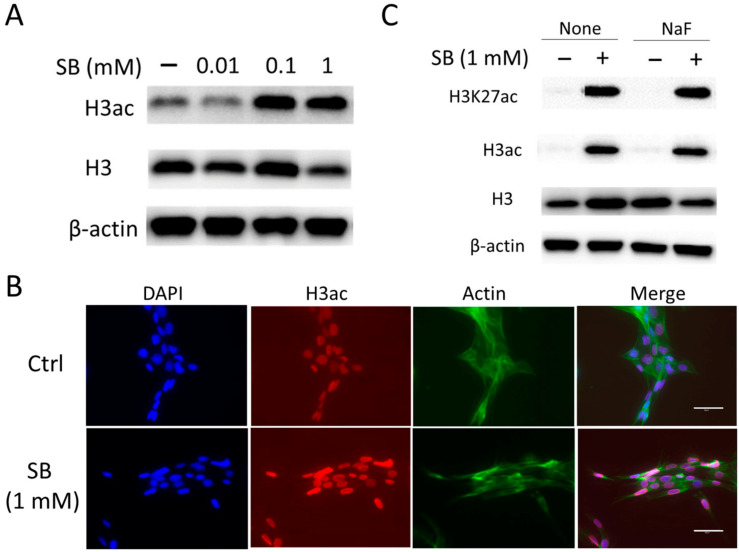
Increase in histone acetylation (H3ac and H3K27ac) via sodium butyrate in LS8 cells. LS8 cells were treated with 1 mM SB with or without 5 mM NaF for 24 h. (**A**) H3ac (17 kDa) levels were detected via WB. SB increased the protein level of H3ac dose-dependently. β-Actin (44 kDa) was used as a loading control. Representative images are shown. Quantification and statistical analyses of relative protein levels are shown in Supplementary Figure S2. (**B**) SB treatment for 24 h increased the signal of H3ac in nuclei compared to control (Ctrl). Nucleus (DAPI; blue), H3ac (red), and Actin (green) were detected via immunofluorescence. Representative images are shown. Scale bars; 50 μm. (**C**) H3K27ac (17 kDa) expressions were detected via WB. SB increased the protein levels of H3ac and H3K27ac with or without fluoride. β-Actin (44 kDa) was used as a loading control. Representative images are shown. Quantification and statistical analyses of relative protein levels are shown in Supplementary Figure S3.

Sodium butyrate (SB) is known to inhibit activity of HDACs causing hyperacetylation of histones [25,28]. LS8 cells were treated with 1 mM SB for 24 h (the concentration of SB was determined based on previous studies [29,30]). HDAC activity and histone acetylation (H3ac and H3K27ac) were detected via Western blot and immunofluorescent. SB significantly decreased p-HDAC2 (*p <* 0.01) and p-HDAC7 (*p <* 0.05) levels. It also decreased p-SIRT1 (*p <* 0.05) but not p-HDAC3 levels when compared to untreated controls (Figure 5 and Figure S1B). SB significantly increased H3ac in LS8 cells in a dose-dependent manner (*p <* 0.01) (Figure 6A and Figure S2). Immunofluorescent staining revealed that SB increased H3ac in LS8 nuclei compared to controls (Figure 6B). Next, SB effects on fluoride treatment were evaluated. LS8 cells were treated with 5 mM NaF for 24 h with or without 1 mM SB. SB significantly increased H3ac and H3K27ac levels in the presence or absence of NaF treatment (Figure 6C and Figure S3). 

### 2.6. Addition of SB Increases the mRNA Levels of Bcl2 and Bad Compared to Fluoride Treatment Alone

**Figure 7:**
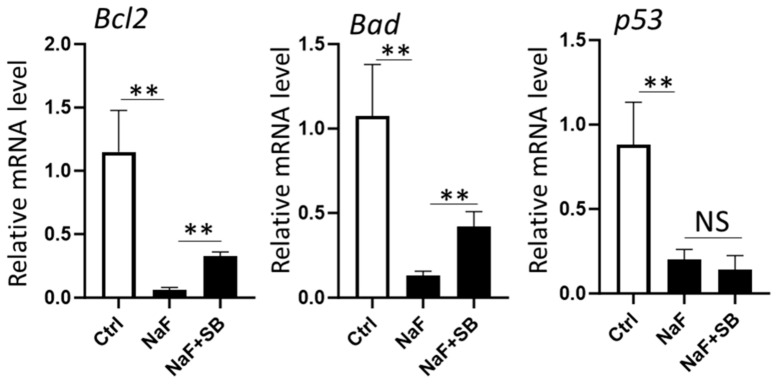
The effect of sodium butyrate on identified genes’ mRNAs which are associated with histone H3K27 deacetylation via fluoride treatment. LS8 cells were treated with 1 mM SB for 1 h prior to 5 mM NaF treatment for 24 h. qPCR results showed that addition of SB significantly increased mRNA levels of *Bcl2* and *Bad* compared to NaF alone, but *p53* mRNA level was not changed with SB addition. Data are presented as means ± SD. (N = 4/group). ** *p <* 0.01, NS; no significant differences.

To investigate the roles of HDACs in fluoride-mediated gene suppression of *Bcl2, Bad*, *and p53* (Figure 2), we assessed if SB altered the mRNA levels of these genes. LS8 cells were treated with 5 mM NaF for 24 h with or without 1 mM SB. The addition of SB significantly increased the mRNA levels of *Bcl2* and *Bad* compared to fluoride exposure alone (*p* < 0.01) but did not alter the mRNA level of *p53* (Figure 7). These results suggest that HDAC activity and histone acetylation are involved in fluoride-mediated *Bcl2* and *Bad* gene suppression, while *p53* gene suppression via fluoride treatment appears to be regulated in an HDAC-independent fashion. Although SB increased histone acetylation (H3ac and H3K27ac) without NaF (Figure 6), SB alone did not alter mRNA levels of *p21, Mdm2, Bax, Bcl2, Bad*, or *p53* when compared to controls (Figure S4).

### 2.7. Addition of SB Suppresses Fluoride-Mediated Apoptosis by Decreasing the mRNA Levels of p21, Mdm2, and the Bax/Bcl2 Ratio

**Figure 8:**
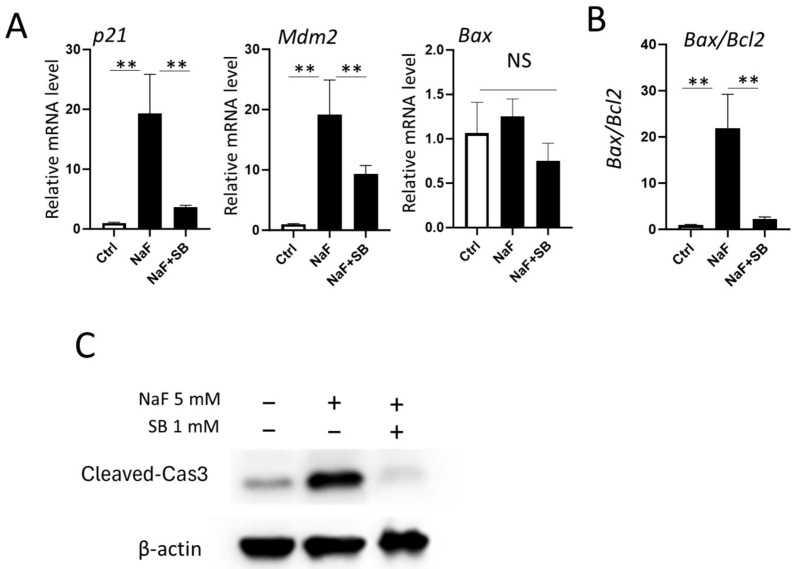
Sodium butyrate suppresses fluoride-mediated apoptosis in LS8 cells. LS8 cells were treated with 1 mM SB for 1 h prior to 5 mM NaF for 24 h. (**A**) qPCR results showed that addition of SB significantly decreased the mRNA levels of *p21* and *Mdm2* but did not change *Bax* mRNA. (**B**) SB addition suppressed the mRNA ratio of *Bax*/*Bcl2* compared to NaF treatment alone. Data are presented as means ± SD. (N = 4/group). ** *p <* 0.01, NS; no significant differences. (**C**) Cleaved-caspase-3 (17 kDa) was detected via WB. SB with fluoride significantly decreased the protein level of cleaved-caspase-3 compared to fluoride treatment alone. β-Actin (44 kDa) was used as a loading control. Representative images are shown. Quantification and statistical analyses of relative protein levels are shown in Supplementary Figure S5.

Since SB enhanced H3ac and H3K27ac formation with fluoride treatment (Figure 6C), we expected that the addition of SB would potentiate the expression of *p21*, *Mdm2*, and *Bax* compared to treatment with fluoride alone. However, intriguingly, SB significantly downregulated fluoride-induced *p21* and *Mdm2* mRNAs compared to just fluoride treatment (*p <* 0.01) (Figure 8A). This suggests that other signaling pathways are involved in the SB-mediated gene suppression of *p21* and *Mdm2*. The addition of SB suppressed the mRNA ratio of *Bax/Bcl2* compared to fluoride alone (*p* < 0.01) (Figure 8B) by increasing *Bcl2* mRNA levels. (Figure 7). This was accompanied by the suppression of fluoride-mediated apoptosis. The addition of SB significantly decreased the fluoride-induced cleaved-caspase 3 formation compared to fluoride alone (*p <* 0.01) (Figure 8C and Figure S5).

## 3. Discussion 

Epigenetic modifications, including DNA methylation, histone acetylation, and non-coding RNAs, regulate gene expression without altering DNA sequence [13,14]. A recent systematic review has shown the effect of fluoride on epigenetic modification in various tissues, including bone, brain, liver, kidney, and testes [31]. However, there are limited studies on epigenetic mechanisms in dental fluorosis. To our knowledge, this is the first study to report on fluoride-mediated regulation of histone acetylation in dental fluorosis.

**Figure 9:**
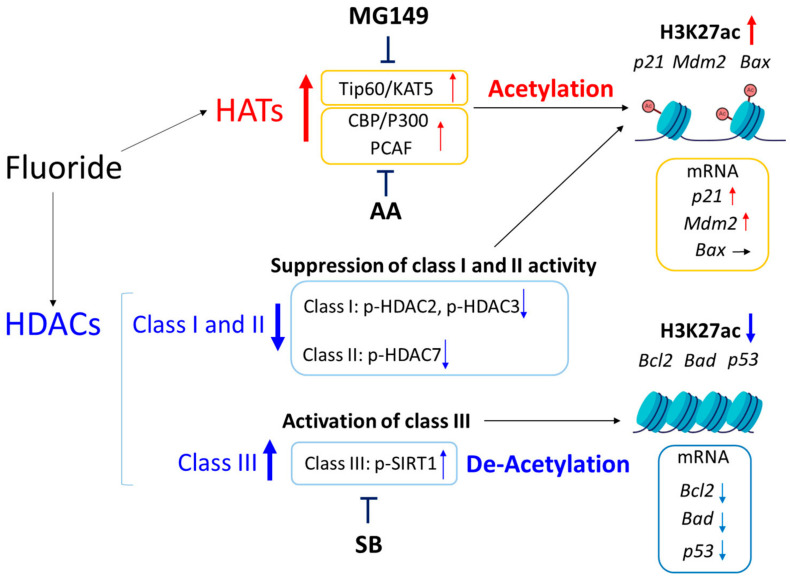
Schema of fluoride-mediated gene alterations via H3K27 acetylation in LS8 cells. Fluoride activates HATs and upregulates acetylation of H3K27 near transcription start sites (TSS) of *p21*, *Mdm2*, and *Bax* genes. The mRNA levels of *p21* and *Mdm2* are increased, but *Bax* mRNA levels are not changed. HAT inhibitors AA (CBP/P300 and PCAF inhibitor) or MG149 (Tip60/KAT5 inhibitor) suppress the transcription of *p21* and *Mdm2* associated with acetylation of H3K27. Fluoride suppresses active form class I HDAC (p-HDAC2 and p-HDAC3) and class II HDAC (p-HDAC7), which is involved in the increase in histone acetylation. On the other hand, fluoride activates class III HDAC (p-SIRT1) which is involved in deacetylation of H3K27 to suppress mRNA levels of *Bcl2*, *Bad*, and *p53*. HDAC inhibitor sodium butyrate (SB) suppresses fluoride-mediated SIRT1 activation to increase the mRNA levels of *Bcl2* and *Bad*. Up arrows: increase, Down arrows: decrease, Right arrows: no change.

Figure 9 shows a schematic diagram illustrating the fluoride-mediated epigenetic gene alterations in LS8 cells. ChIP-seq results demonstrated that fluoride increased H3K27ac levels in the promoters of *Bax*, *Mdm2* and *p21*, while reducing H3K27ac in the promoter regions of *Bcl2, Bad*, and *p53*. Fluoride-mediated histone acetyltransferase (HAT) activation and histone deacetylase (HDAC) (class I and II) suppression could contribute to histone acetylation, while fluoride-mediated SIRT1 (class III HDAC) is likely a contributing factor for histone deacetylation. mRNA levels of *p21, Mdm2, Bcl2, Bad, and p53* were associated with H3K27ac levels in their promoter regions. However, *Bax* mRNA levels were not in agreement with H3K27ac status, suggesting that *Bax* is regulated independently of fluoride-mediated H3K27ac. *Bax* mRNA expression is regulated by various mechanisms [32], including other epigenetic modifications. A previous study has shown that reduced *Bax* gene expression is associated with the suppression of H4K16ac and the increase in H4K20Me3 histone methylation in senescent human diploid fibroblasts [33]. Additionally, fluoride has been reported to increase the DNA methylation of Neuronatin gene *NNAT*, leading to decreased expression and disrupted glucose transport in porcine oocytes in vitro [34]. Fluoride induces DNA hypermethylation at the promoter regions of *Col1a1* and reduces its gene expression in fluoride treated mice [35]. Fluoride also induces histone trimethylation of H3K9 and H3K27 at the promoter regions of *TGFBR2* and *SMAD*, which contributes to the development of skeletal fluorosis in vitro [36]. Thus, the discrepancy between fluoride-mediated H3K27ac status and *Bax* mRNA expression may be due to other factors, including other epigenetic modifications at different histone lysine residues such as trimethylations or DNA methylation.

Fluoride increased the mRNA levels of *p21* and *Mdm2*, and this effect was inhibited via HAT inhibitors in LS8 cells. This suggests that HAT plays a crucial role in the transcriptional regulation of *p21* and *Mdm2*, which is associated with H3K27 acetylation in dental fluorosis. When treated with fluoride, our results show that *p21* and *Mdm2* are differentially regulated by CBP/p300, PCAF, and Tip60/KAT5. Also, in contrast to MG149 (Tip60/KAT5 inhibitor), which suppressed fluoride-induced mRNAs of both *p21* and *Mdm2*, AA (CBP/p300 and PCAF inhibitor) did not change *Mdm2* expression. This indicates that Tip60/KAT5 is involved in the regulation of both *p21* and *Mdm2* in response to fluoride, whereas CBP/p300 and PCAF are involved only in the regulation of *p21* gene expression but not *Mdm2*.

Previously, we have reported that fluoride activates SIRT1 (class III HDAC) as an adaptive response against fluoride toxicity in ameloblasts in vitro and in rodents in vivo [9]. However, fluoride effects on class I and II HDACs are not well characterized during amelogenesis. In the present study, we demonstrate that fluoride suppresses the activities of class I HDACs (HDAC2 and HDAC3) and class II HDAC (HDAC7) in LS8 cells (Figure 4). NaF also suppressed total HDACs (Supplementary Figure S6), suggesting that fluoride treatment may inhibit HDACs at transcriptional and/or translational levels. Previous studies demonstrated that inhibition of HDAC2 and HDAC3 increased *p21* expression in human colon cancer [37]. Moreover, HDAC2 knockdown in mouse neuronal cells (HT-22) upregulates *p21* mRNA expression [38]. These studies are concordant with our results, suggesting that fluoride-mediated suppression of class I and II HDACs contributes to the upregulation of *p21* and *Mdm2* mRNA in ameloblasts. SIRT1 regulates gene expression by deacetylating transcription factors, including p53, Forkhead box-O (FOXO), and NF-kB, thereby modifying their activity directly [39]. So, fluoride-mediated SIRT1 activation likely contributes to the reduction in *Bcl2*, *Bad*, and *p53* mRNA. Although sodium butyrate (SB) inhibited SIRT1 activity and increased H3 and H3K27 acetylation, fluoride-mediated gene suppression of *Bcl2* and *Bad* was partially reversed by SB treatment. Moreover, fluoride-mediated *p53* suppression was not reversed by SB treatment. These results suggest that in addition to the activation of SIRT1, other factors are likely involved in fluoride-mediated mRNA suppression of *Bcl2*, *Bad*, and *p53*. For example, fluoride-mediated DNA methylation and histone trimethylation [34,36] could be involved. SB treatment induced hyperacetylation of H3 and H3K27 with or without fluoride. Consequently, we expected that SB would augment the fluoride-induced *Mdm2* and *p21* mRNA expression. However, intriguingly, the addition of SB significantly downregulated fluoride-induced *p21* and *Mdm2* mRNAs compared to fluoride treatment alone. In addition to being an HDAC inhibitor, SB is linked to DNA methylation changes in eukaryotic cells [40,41]. Therefore, the mRNA repression of *Mdm2* and *p21* via SB may be caused by increased DNA methylation. 

SB’s biological effects vary depending on cell and tissue types. A previous study has demonstrated that SB inhibits SIRT3 to promote apoptosis in cancer cells [25]. In the present study, SB decreased the *Bax/Bcl2* mRNA ratio in the presence of fluoride, which attenuated fluoride-mediated apoptosis by reducing cleaved-caspase-3 formation in LS8 cells. Concordant with our results, recent studies have shown that SB protects rats against skeletal fluorosis by regulating bone homeostasis and serum metabolism via arginine and proline pathways [42]. SB ameliorates fluoride-induced neurotoxicity by activating the PI3K/AKT pathway to promote glycolysis metabolism [43]. We previously demonstrated that curcumin attenuates fluoride-mediated apoptosis via activation of AKT in LS8 cells [44]. These studies indicate that in addition to HDAC regulation, activation of AKT via SB may contribute to the attenuation of fluoride toxicity in LS8 cells.

In this study, we demonstrated for the first time that fluoride regulates gene expression through the modulation of H3K27 acetylation in LS8 cells. Further research is required to understand the mechanisms of fluoride-mediated epigenetic regulation in amelogenesis. Our findings lay the foundation for the development of novel preventive or therapeutic strategies targeting epigenetic factors to mitigate fluoride toxicity.

## 4. Materials and Methods

### 4.1. Cell Culture

LS8 cells derived from mouse enamel organ epithelia [45] were grown in alpha minimal essential medium with GlutaMAX (Thermo Fisher Scientific, Waltham, MA, USA) supplemented with fetal bovine serum (10%) and sodium pyruvate (1 mM). Cells were treated with sodium fluoride (NaF; 0, 1 mM, 3 mM, and 5 mM, Thermo Fisher Scientific) with or without anacardic acid (AA; inhibitor of CBP/p300 and PCAF), MG149 (Inhibitor of Tip60/KAT5), and sodium butyrate (SB; inhibitor of histone deacetylase, HDAC) as indicated (Selleck Chemicals, Houston, TX, USA). NaF was freshly dissolved in a medium at the time of use. AA and MG149 were formulated in a vehicle of dimethyl sulfoxide (DMSO; MilliporeSigma, Burlington, MA, USA), and SB was formulated in a vehicle of distilled water. Moreover, 0.1% DMSO and 0.04% DMSO were used as vehicle control for AA and MG149, respectively. NaF concentrations were used based on previous studies, which demonstrated that 5 mM NaF induces biological effects on LS8 cells in vitro [9,10,11,19,44]. Histone acetyltransferase (HAT) inhibitors (AA and MG149) [19] and sodium butyrate (SB) concentrations were used based on previous studies [29,30].

### 4.2. ChIP-Sequence

LS8 cells were (1 × 10^6^ cells/10 cm dish) cultured overnight and treated with or without NaF (5 mM) for 24 h. Four dishes of cells treated with or without NaF were combined and used as one independent replicate (4 × 10^6^ cells/replicate). After that, cells were subjected to chromatin isolation followed by immunoprecipitation and DNA purification using the iDeal ChIP-seq Kit for Histones (Diagenode, Denville, NJ, USA) according to the manufacturer’s instructions. In brief, cells were cross-linked with 1% formaldehyde and the crosslinking was stopped by adding glycine. Cells were subsequently lysed, and the isolated chromatin was sheared in the kit-provided lysis buffer via sonication (30 s ON, 30 s OFF, 30 cycles) using a Bioruptor^®®^ Pico (Diagenode). The diluted sheared chromatins (approximately 150–300 bp-long DNA fragments) were incubated with ChIP-grade antibodies overnight at 4 °C on a rotator and immunoprecipitated with magnetic beads. Antibodies used for ChIP assays were obtained from Cell Signaling Technology (Danvers, MA, USA): anti-Histone 3 antibody (4620S) and anti-H3K27ac antibody (8173S). According to the company’s instructions, antibodies (1:50 dilution for 10 ug of chromatin, approximately 4 × 10^6^ cells) were used for each independent replicate. Isolated magnetic beads were extensively washed, followed by resuspension into the kit-provided buffer and incubation overnight at 4 °C to allow for the decrosslinking of the captured DNA. DNA was purified using the IPure kit v2 (Diagenode). The input ChIP-enriched DNA fragments were analyzed on an Agilent 2200 TapeStation System (Agilent Technologies, Miami, FL, USA) to quantify and assess the DNA fragment sizes. Five independent experiments were performed. Pooled samples from five independent experiments were used for library preparation. 

ChIP-seq libraries were prepared using the TruSeq ChIP Library Preparation Kit (Illumina, San Diego, CA, USA). Briefly, 5–10 ng of input ChIP DNA fragments with a size range of 200–800 bp were end repaired by removing the 3’ overhangs and filling the 5’ overhangs using the DNA polymerase. Following purification, the blunt-ended fragments had the addition of a single ‘A’ base and ligation of the adaptors that contained a ‘T’ base overhang. The products were purified and enriched with PCR to create the final ChIP-seq library. The prepared library was examined via a bioanalyzer and Qubit (Thermo Fisher Scientific) to test library quality and quantity, respectively. Sequencing was performed on the Nextseq500 (Illumina) using the High Output v2.5 kit, with a 75-cycle single-read protocol. BCL files generated via the Nextseq500 were converted to FASTQ files for the downstream analysis. The FASTQ files had quality scores above 35 on a scale of 1 to 40, with 20 to 30 million reads obtained per sample. Reads passing the quality control were aligned to the reference genome starting with Bowtie2 or BWA aligner. Samtools (version 1.6) was used to convert SAM files to the sorted BAM. The generated BAM files in a comparative setup were imported to the MACS2 (https://hbctraining.github.io/Intro-to-ChIPseq/lessons/05_peak_calling_macs.html, accessed on 3 March 2024) for peak calling to output the genomic locations of enriched peaks with -log10 q values at a threshold of 10–2.5 and annotated using java PeakAnnotator (version 1.4) in a peak-calling bed format processed with bedtools. The enriched peaks of each sample in bigwig files with the genomic annotations were shown in the Integrative Genomics Viewer (IGV, https://software.broadinstitute.org/software/igv/) (Version 2.17.3, accessed on 3 March 2024) and plotted. (Bioinformatics and HPC Shared Resource Integrated Genomics Facility Affiliation Georgia Cancer Center, Augusta University, Augusta, GA, USA).

### 4.3. Real Time PCR

Real-time PCR was performed as described previously [44]. Briefly, for total RNA extraction, Direct-zol RNA MiniPrep (Zymo Research Corp, Irvine, CA, USA) was used. The cDNA was synthesized using an iScript cDNA Synthesis Kit (Bio-Rad Laboratories Inc, Hercules, CA, USA) on a C1000 Touch thermal cycler (Bio-Rad). The cDNA was subjected to qPCR amplification on QuantStudio 3 (Thermo Fisher Scientific). Primer sequences are presented in Supplementary Table S1. Beta-2-microglobulin (*B2m*), which had consistent expression with experimental treatments, was used as an internal reference control gene. Data were analyzed using the 2^-ΔΔCT^ method [46]. Four to five independent experiments were performed.

### 4.4. Western Blotting

LS8 cells (1 × 10^6^ cells/dish) were seeded in 10 cm dishes and cultured for 24 h. After cells were lysed, total proteins were extracted with RIPA buffer and 1% Halt protease inhibitor (Thermo Fisher Scientific). Equal amounts of protein samples were subjected to Western blot analysis as described previously [47]. The following antibodies were used. Primary Ab: rabbit anti-phospho-HDAC2 (Ser394) (#69238), rabbit anti-phospho-HDAC3 (Ser424) (#3815), rabbit anti-phospho-HDAC7 (Ser155) (#3443), rabbit anti-acetyl-Histone H3 (K27) (#8173), mouse anti-Histone H3 (#14269), mouse anti-α-tubulin (#3873), mouse anti-β-actin (#3700) (1:1000, Cell Signaling Technology, Boston, MA, USA), rabbit anti-Histone H3ac (pan-acetyl) (PA5-116785) (1:1000, Thermo Fisher Scientific), and rabbit anti-phospho-SIRT1 (Ser47) (BS-3393R) (1:1000, Bioss, Woburn, MA, USA). Secondary Ab: HRP-conjugated anti-rabbit IgG (#7074) or HRP-conjugated anti-mouse IgG (#7076) (1:5000) (Cell Signaling Technology). Enhanced chemiluminescence was performed and the signal was detected via an Azure 400-Fluorescent Western Blot Imager (Azure Systems, Dublin, CA, USA). Representative images are shown in the results section. Band densities were quantified using ImageJ.JS (ImJoy) (ImageJ 1.53m, NIH, USA). Relative protein expression data are presented as means ± SD. At least three independent assays were analyzed for each experiment.

### 4.5. Immunofluorescence

LS8 cells were cultured on micro cover glasses (VWR, Radnor, PA, USA) in 24-well plates and treated with NaF for 24 h with/without SB. After that, cells were fixed in 4% PFA (5 min), followed by incubation in 0.3% Triton-X-100 (10 min) and 5% BSA blocking buffer (1 h) at RT. Next, cells were incubated with rabbit anti-Histone H3ac (pan-acetyl) (PA5-116785) (1:200, Thermo Fisher Scientific) or rabbit anti-acetyl-Histone H3 (K27) (#8173) (1:200, Cell Signaling Technology) overnight at 4 °C. The cells were washed with PBS and incubated at RT with the secondary antibodies AlexaFluor 594-conjugated goat anti-rabbit IgG (Thermo Fisher Scientific) (1 h) and CellMask™ Green Actin Tracking Stain (Thermo Fisher Scientific) (15 min) and were mounted with Fluoromount-GTM medium with DAPI (Thermo Fisher Scientific) on glass slides. The cells were analyzed using fluorescence microscopy (EVOS M5000, Thermo Fisher Scientific). At least three independent assays for each experiment were performed, and representative images are shown.

### 4.6. Statistical Analysis

Data were analyzed using Student’s *t*-test or one-way analysis of variance (ANOVA) with the post hoc analysis using Tukey’s multiple comparisons test using Graph Pad Prism 10. Significance was assessed at *p <* 0.05.

## 5. Conclusions

In the present study, we report the first evidence that fluoride alters gene expressions via histone acetylation in ameloblast-like LS8 cells in vitro. These findings suggest that using agents that promote epigenetic modification may be a potential strategy for the prevention or mitigation of dental fluorosis. Further studies are required to characterize the fluoride-mediated epigenetic mechanisms involved in enamel development.

## Figures and Tables

**Table 1:** Location of H3K27 acetylation peaks in genes acetylated or deacetylated via fluoride treatment in LS8 cells.

	Histone	Chromosome	PeakStart	PeakEnd	Distance	GeneStart	GeneEnd	ClosestTSS_ID	Symbol	Strand	Enrichment	Qvalues
acetylated	H3K27	chr7	52,721,282	52,722,261	373	52,717,232	52,722,145	ENSMUST00000169539	*Bax*	−	2.65055	4.98461
		chr10	117,146,295	117,147,436	212	117,125,951	117,147,078	ENSMUST00000155285	*Mdm2*	−	2.47539	4.63473
		chr17	29,230,713	29,231,971	625	29,230,717	29,237,667	ENSMUST00000023829	*p21*	+	2.31322	5.8589
De-acetylated	H3K27	chr1	108,610,964	108,611,451	−356	108,434,755	108,610,851	ENSMUST00000112751	*Bcl2*	−	2.49999	1.6118
		chr1	108,610,216	108,610,628	429	108,434,755	108,610,851	ENSMUST00000112751	*Bcl2*	−	2.84654	1.61343
		chr11	69,394,210	69,394,667	531	69,393,907	69,405,375	ENSMUST00000108657	*Trp53*	+	1.63639	2.34386
		chr19	7,016,341	7,017,061	−112	7,016,813	7,026,382	ENSMUST00000113426	*Bad*	+	2.19519	3.62331

## Data Availability

The datasets generated and/or analyzed are partly available within this article as supplemental files; other raw data are available from the corresponding author on reasonable request.

## Supplementary Materials

The following supporting information can be downloaded at: https://www.mdpi.com/article/10.3390/ijms25179600/s1.

## References

[1] U.S. Department of Health, Human Services Federal Panel on Community Water Fluoridation U.S (2015). Public Health Service Recommendation for Fluoride Concentration in Drinking Water for the Prevention of Dental Caries. Public Health Rep..

[2] Asawa K., Singh A., Bhat N., Tak M., Shinde K., Jain S. (2015). Association of Temporomandibular Joint Signs & Symptoms with Dental Fluorosis & Skeletal Manifestations in Endemic Fluoride Areas of Dungarpur District, Rajasthan, India. J. Clin. Diagn. Res..

[3] Vasisth D., Mehra P., Yadav L., Kumari V., Bhatia U., Garg R. (2024). Fluoride and its Implications on Oral Health: A Review. J. Pharm. Bioallied Sci..

[4] DenBesten P., Wu L. (2011). Chronic fluoride toxicity: Dental fluorosis. Monogr Oral Sci..

[5] McKay F.S. (1952). The Study of Mottled Enamel (Dental Fluorosis). J. Am. Dent. Assoc..

[6] American Public Health Association (1933). MOTTLED ENAMEL. Am. J. Public Health Nations Heath.

[7] Smith C., Issid M., Margolis H., Moreno E. (1996). Developmental Changes in the PH of Enamel Fluid and Its Effects On Matrix-Resident Proteinases. Adv. Dent. Res..

[8] Sharma R., Tsuchiya M., Skobe Z., Tannous B.A., Bartlett J.D. (2010). The Acid Test of Fluoride: How pH Modulates Toxicity. PLoS ONE.

[9] Suzuki M., Bartlett J.D. (2013). Sirtuin1 and autophagy protect cells from fluoride-induced cell stress. Biochim. Biophys. Acta.

[10] Suzuki M., Bandoski C., Bartlett J.D. (2015). Fluoride induces oxidative damage and SIRT1/autophagy through ROS-mediated JNK signaling. Free Radic. Biol. Med..

[11] Suzuki M., Ikeda A., Bartlett J.D. (2017). Sirt1 overexpression suppresses fluoride-induced p53 acetylation to alleviate fluoride toxicity in ameloblasts responsible for enamel formation. Arch. Toxicol..

[12] Berger S.L., Kouzarides T., Shiekhattar R., Shilatifard A. (2009). An operational definition of epigenetics. Genes Dev..

[13] Handy D.E., Castro R., Loscalzo J. (2011). Epigenetic modifications: Basic mechanisms and role in cardiovascular disease. Circulation.

[14] Jenuwein T., Allis C.D. (2001). Translating the Histone Code. Science.

[15] Singh R., Rathore A.S., Dilnashin H., Keshri P.K., Gupta N.K., Prakash S.A.S., Zahra W., Singh S., Singh S.P. (2024). HAT and HDAC: Enzyme with Contradictory Action in Neurodegenerative Diseases. Molecular Neurobiology.

[16] Voss A.K., Thomas T. (2018). Histone Lysine and Genomic Targets of Histone Acetyltransferases in Mammals. BioEssays.

[17] Tomasiak P., Janisiak J., Rogińska D., Perużyńska M., Machaliński B., Tarnowski M. (2023). Garcinol and Anacardic Acid, Natural Inhibitors of Histone Acetyltransferases, Inhibit Rhabdomyosarcoma Growth and Proliferation. Molecules.

[18] Fahmy S.H., Jungbluth H., Jepsen S., Winter J. (2023). Effects of histone acetyltransferase (HAT) and histone deacetylase (HDAC) inhibitors on proliferative, differentiative, and regenerative functions of Toll-like receptor 2 (TLR-2)-stimulated human dental pulp cells (hDPCs). Clin. Oral Investig..

[19] Deng H., Fujiwara N., Cui H., Whitford G.M., Bartlett J.D., Suzuki M. (2020). Histone acetyltransferase promotes fluoride toxicity in LS8 cells. Chemosphere.

[20] Chen H.P., Zhao Y.T., Zhao T.C. (2015). Histone Deacetylases and Mechanisms of Regulation of Gene Expression. Crit. Rev. Oncog..

[21] Milazzo G., Mercatelli D., Di Muzio G., Triboli L., De Rosa P., Perini G., Giorgi F.M. (2020). Histone Deacetylases (HDACs): Evolution, Specificity, Role in Transcriptional Complexes, and Pharmacological Actionability. Genes.

[22] I Razick D., Akhtar M., Wen J., Alam M., Dean N., Karabala M., Ansari U., Ansari Z., Tabaie E., Siddiqui S. (2023). The Role of Sirtuin 1 (SIRT1) in Neurodegeneration. Cureus.

[23] Elfadil O.M., Mundi M.S., Abdelmagid M.G., Patel A., Patel N., Martindale R. (2023). Butyrate: More Than a Short Chain Fatty Acid. Curr. Nutr. Rep..

[24] Patnala R., Arumugam T.V., Gupta N., Dheen S.T. (2017). HDAC Inhibitor Sodium Butyrate-Mediated Epigenetic Regulation Enhances Neuroprotective Function of Microglia During Ischemic Stroke. Mol. Neurobiol..

[25] Xu S., Liu C.-X., Xu W., Huang L., Zhao J.-Y., Zhao S.-M. (2017). Butyrate induces apoptosis by activating PDC and inhibiting complex I through SIRT3 inactivation. Signal Transduct. Target. Ther..

[26] Cahyadi A., Ugrasena I.D.G., Andarsini M.R., Larasati M.C.S., Aryati A., Arumsari D.K. (2022). Relationship between Bax and Bcl-2 Protein Expression and Outcome of Induction Phase Chemotherapy in Pediatric Acute Lymphoblastic Leukemia. Asian Pac. J. Cancer Prev..

[27] Bahl S., Seto E. (2021). Regulation of histone deacetylase activities and functions by phosphorylation and its physiological relevance. Cell. Mol. Life Sci..

[28] Candido E. (1978). Sodium butyrate inhibits histone deacetylation in cultured cells. Cell.

[29] Liu J., Wang Y., Wu Y., Ni B., Liang Z. (2014). Sodium Butyrate Promotes the Differentiation of Rat Bone Marrow Mesenchymal Stem Cells to Smooth Muscle Cells through Histone Acetylation. PLoS ONE.

[30] Zhang K., Hussain T., Wang J., Li M., Wang W., Ma X., Liao Y., Yao J., Song Y., Liang Z. (2020). Sodium Butyrate Abrogates the Growth and Pathogenesis of Mycobacterium bovis via Regulation of Cathelicidin (LL37) Expression and NF-κB Signaling. Front. Microbiol..

[31] Balasubramanian S., Perumal E. (2022). A systematic review on fluoride-induced epigenetic toxicity in mammals. Crit. Rev. Toxicol..

[32] Casamassimi A., Casamassimi A., Ciccodicola A., Ciccodicola A. (2019). Transcriptional Regulation: Molecules, Involved Mechanisms, and Misregulation. Int. J. Mol. Sci..

[33] Sanders Y.Y., Liu H., Zhang X., Hecker L., Bernard K., Desai L., Liu G., Thannickal V.J. (2013). Histone Modifications in Senescence-Associated Resistance to Apoptosis by Oxidative Stress. Redox Biol..

[34] Liu X., Nie Z., Gao Y., Chen L., Yin S., Zhang X., Hao C., Miao Y. (2018). Sodium fluoride disturbs DNA methylation of *NNAT* and declines oocyte quality by impairing glucose transport in porcine oocytes. Environ. Mol. Mutagen..

[35] Bhowmik A.D., Das T., Chattopadhyay A. (2023). Chronic exposure to environmentally relevant concentration of fluoride impairs osteoblast’s collagen synthesis and matrix mineralization: Involvement of epigenetic regulation in skeletal fluorosis. Environ. Res..

[36] Daiwile A.P., Sivanesan S., Tarale P., Naoghare P.K., Bafana A., Parmar D., Kannan K. (2018). Role of fluoride induced histone trimethylation in development of skeletal fluorosis. Environ. Toxicol. Pharmacol..

[37] Wilson A.J., Byun D.-S., Popova N., Murray L.B., L’Italien K., Sowa Y., Arango D., Velcich A., Augenlicht L.H., Mariadason J.M. (2006). Histone Deacetylase 3 (HDAC3) and Other Class I HDACs Regulate Colon Cell Maturation and p21 Expression and Are Deregulated in Human Colon Cancer. J. Biol. Chem..

[38] Peng S., Zhao S., Yan F., Cheng J., Huang L., Chen H., Liu Q., Ji X., Yuan Z. (2015). HDAC2 Selectively Regulates FOXO3a-Mediated Gene Transcription during Oxidative Stress-Induced Neuronal Cell Death. J. Neurosci..

[39] Vaziri H., Dessain S.K., Eaton E.N., Imai S.-I., Frye R.A., Pandita T.K., Guarente L., Weinberg R.A. (2001). hSIR2SIRT1 Functions as an NAD-Dependent p53 Deacetylase. Cell.

[40] De Haan J.B., Gevers W., I Parker M. (1986). Effects of sodium butyrate on the synthesis and methylation of DNA in normal cells and their transformed counterparts. Cancer Res..

[41] A Stein R., Riber L. (2023). Epigenetic effects of short-chain fatty acids from the large intestine on host cells. microLife.

[42] Li Y., Yang F., Liu J., Jiang M., Yu Y., Zhou Q., Sun L., Zhang Z., Zhou L., Li Y. (2024). Protective effects of sodium butyrate on fluorosis in rats by regulating bone homeostasis and serum metabolism. Ecotoxicol. Environ. Saf..

[43] Li Y., Wang Z., Li J., Yu Y., Wang Y., Jin X., Dong Y., Liu Q., Duan X., Yan N. (2023). Sodium Butyrate Ameliorates Fluorosis-Induced Neurotoxicity by Regulating Hippocampal Glycolysis In Vivo. Biol. Trace Elem. Res..

[44] Fujiwara N., Whitford G.M., Bartlett J.D., Suzuki M. (2021). Curcumin suppresses cell growth and attenuates fluoride-mediated Caspase-3 activation in ameloblast-like LS8 cells. Environ. Pollut..

[45] Chen L., Couwenhoven R., Hsu D., Luo W., Snead M. (1992). Maintenance of amelogenin gene expression by transformed epithelial cells of mouse enamel organ. Arch. Oral Biol..

[46] Pfaffl M.W. (2001). A new mathematical model for relative quantification in real-time RT-PCR. Nucleic Acids Res..

[47] Fujiwara N., Yamashita S., Okamoto M., Cooley M.A., Ozaki K., Everett E.T., Suzuki M. (2023). Perfluorooctanoic acid-induced cell death via the dual roles of ROS-MAPK/ERK signaling in ameloblast-lineage cells. Ecotoxicol. Environ. Saf..

