# Supplementary material for: Fluoride Alters Gene Expression via Histone H3K27 Acetylation in Ameloblast-like LS8 Cells
Source: Int J Mol Sci. 2024 Sep 4;25(17):9600. doi: 10.3390/ijms25179600 (PMC11395493; doi:10.3390/ijms25179600)
Supplement: Supplementary file 1 [file ijms-25-09600-s001.zip › ijms-3146221-supplementary.pdf]

Supplementary table and figures

Gene	GenBank ID #	5' Primer	3' Primer
p21	NM_007669.5	AATTGGAGTCAGGCGCAGAT	CGAAGAGACAACGGCACACT
Mdm2	NM_010786.4	GTCTGTGTCTACCGAGGGTG	TAAGTGTCGTTTTGCGCTCC
Bax	NM_007527.4	GAGAGGTCTTCTTCCGGGTG	CTGATCAGCTCGGGCACTTT
Bad	NM_001285453.1	TCCTGGGGAGCAACATTCATC	GTACGAACTGTGGCGACTCC
Bcl2	NM_009741.5	TGGATGACTGAGTACCTGAACC	GCCAGGAGAAATCAAACAGAGG
p53	XM_030245922.1	AAGTTCTGTAGCTTCAGTTCATTG	AATGTCTCCTGGCTCAGAGG
B2M	NM_009735.3	GGTCTTTCTGGTGCTTGTCTC	CGTAGCAGTTCAGTATGTTCGG

Table S1 Primer sequences

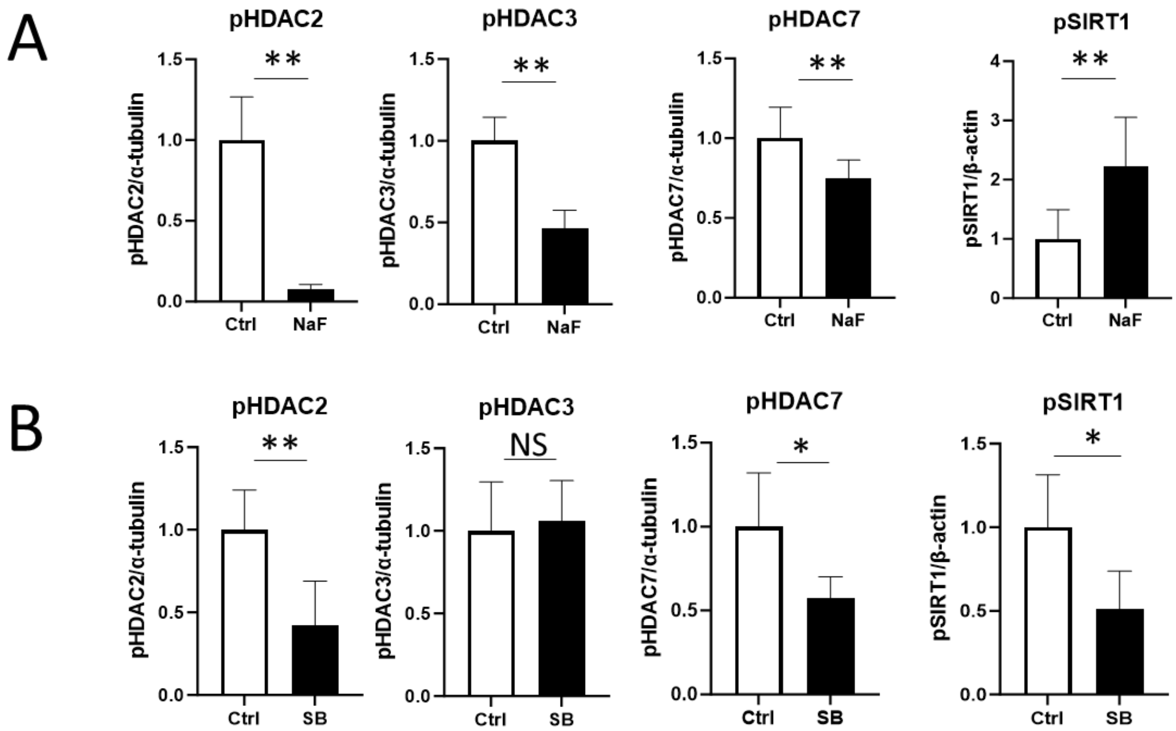

Figure S1. Quantification of relative protein levels of pHDAC2, pHDAC3, pHDAC7 and pSIRT1 in LS8 cells. LS8 cells were treated with (A) 5 mM NaF or (B) 1 mM SB for 24 h. Protein levels were detected by WB and normalized by the loading control; α-tubulin (52 kDa) or β-actin (44 kDa). Graphs show the relative protein levels from five independent experiments. Data are presented as means ± SD. * $p < 0.05$, ** $p < 0.01$, NS; no significant differences.

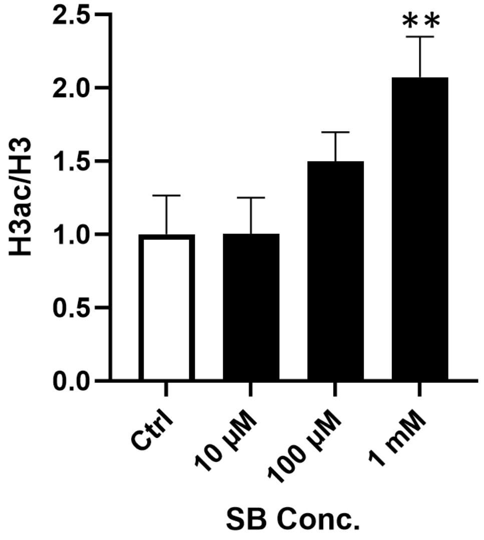

Figure S2. Quantification of relative protein levels of acetylated H3 in LS8 cells.
LS8 cells were treated with SB (0, 10 µM, 100 µM, 1 mM) for 24 h. Acetylated H3 (17 kDa) levels were detected by WB. Protein levels were normalized by the loading control β-actin (44 kDa), and the ratio of H3ac/H3 was calculated. The graph shows the relative protein levels from three independent experiments. Data are presented as means ± SD. ** p < 0.01 vs control.

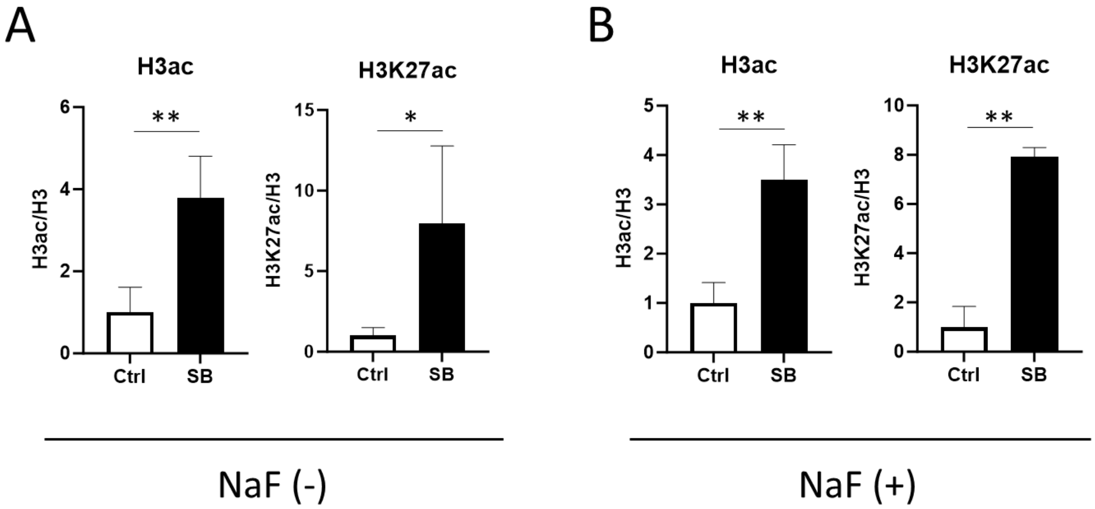

Figure S3. Quantification of relative protein levels of H3ac and H3K27ac in LS8 cells.
LS8 cells were treated with 1 mM SB for 1 h prior to 0 mM or 5 mM NaF for 24 h. H3ac (17 kDa) and H3K27ac (17 kDa) levels were detected by WB. (A) SB without fluoride (B) SB with fluoride treatment. Protein levels were normalized by the loading control β-actin (44 kDa) and the ratio of H3ac/H3 and the ratio of H3K27ac/H3 were calculated. The graphs show the relative protein levels from three to five independent experiments. Data are presented as means ± SD. * p < 0.05, ** p < 0.01

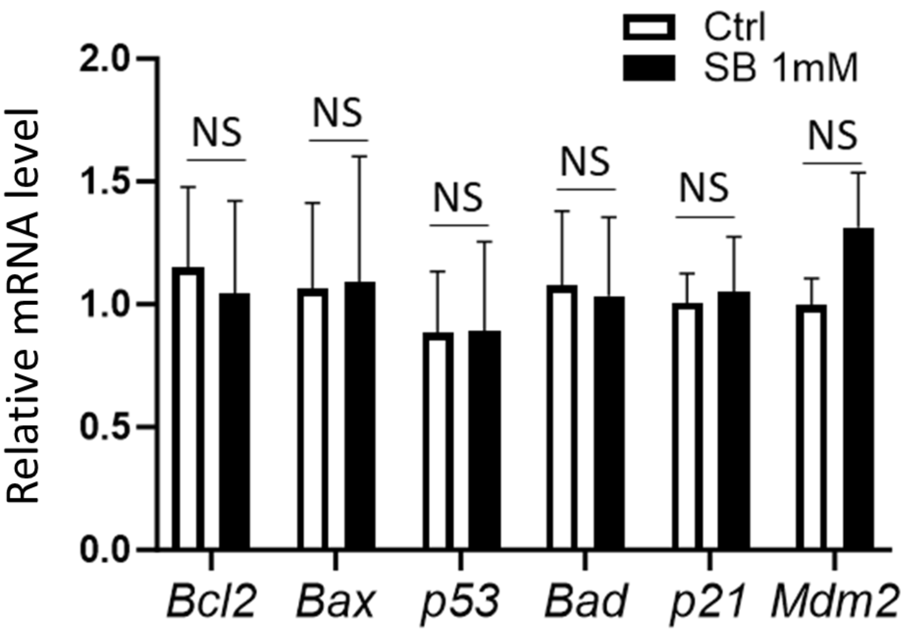

Figure S4. The effect of sodium butyrate on the identified genes mRNAs which are associated with histone H3K27ac altered by fluoride.
LS8 cells were treated with 1 mM SB for 24 h. SB alone did not change mRNA levels of *Bcl2, Bax, p53, Bad, p21* or *Mdm2*. N=6/group. Data are presented as means ± SD. NS; no significant differences.

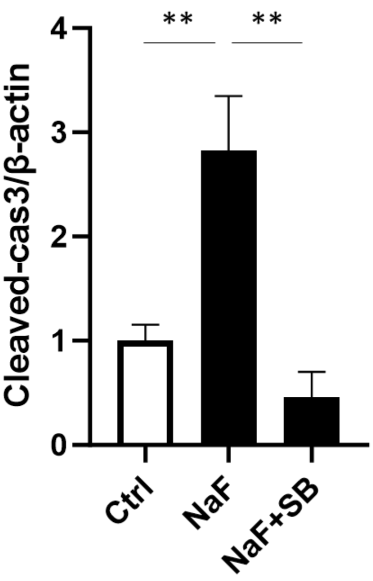

Figure S5. Quantification of relative levels of cleaved-caspase3 in LS8 cells.
LS8 cells were treated with 5 mM NaF with or without 1 mM SB. Cleaved-caspase3 (17 kDa) levels were detected by WB. Protein levels were normalized by the loading control β-actin (44 kDa). Relative protein levels and statistical significance were analyzed. The graph shows the relative protein levels from three independent experiments. Data are presented as means ± SD. ** $p < 0.01$.

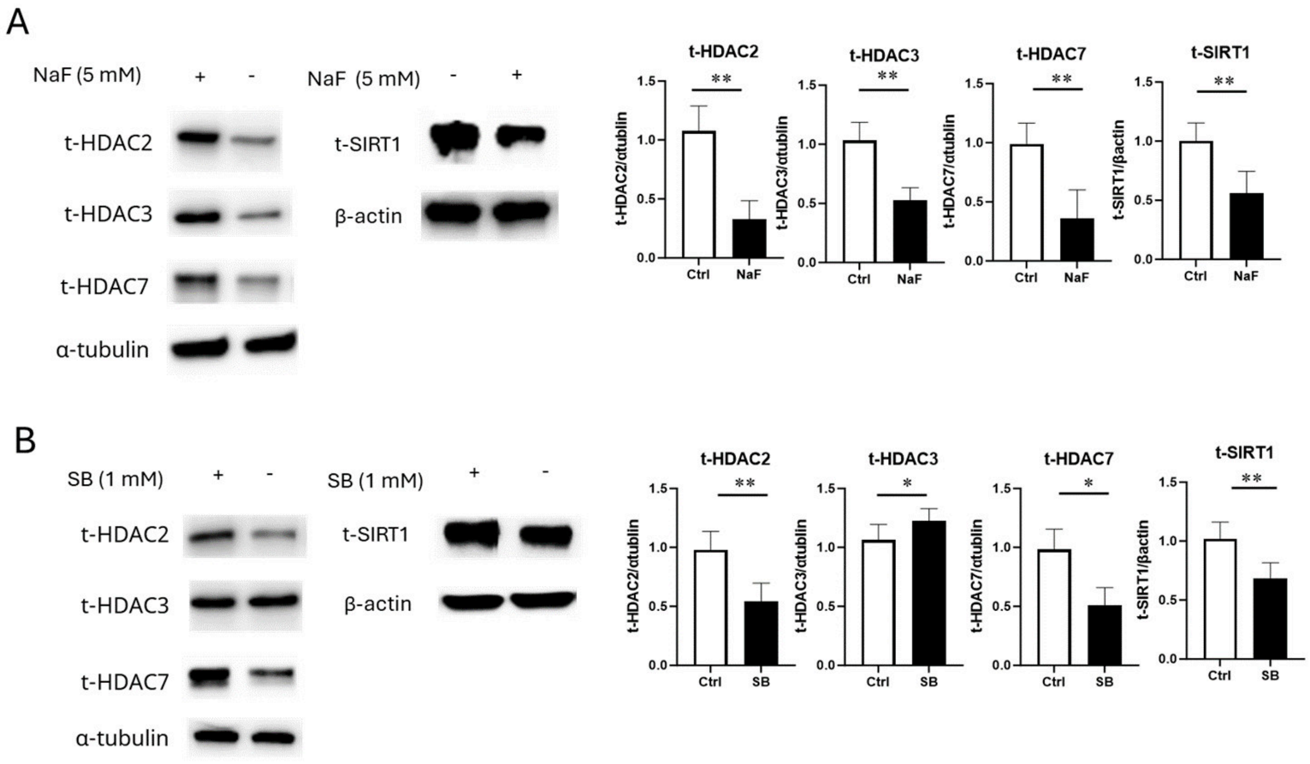

Figure S6. Quantification of relative protein levels of t-HDAC2, t-HDAC3, t-HDAC7 and t-SIRT1 in LS8 cells. LS8 cells were treated with (A) 5 mM NaF or (B) 1 mM SB for 24 h. Protein levels were detected by WB and normalized by the loading control; α-tubulin (52 kDa) or β-actin (44 kDa). Graphs show the relative protein levels from five independent experiments. Data are presented as means ± SD. * $p < 0.05$, ** $p < 0.01$.
