# Supplementary figures and images for: Fluoride Alters Gene Expression via Histone H3K27 Acetylation in Ameloblast-like LS8 Cells
Source: Int J Mol Sci. 2024 Sep 4;25(17):9600. doi: 10.3390/ijms25179600 (PMC11395493; doi:10.3390/ijms25179600)

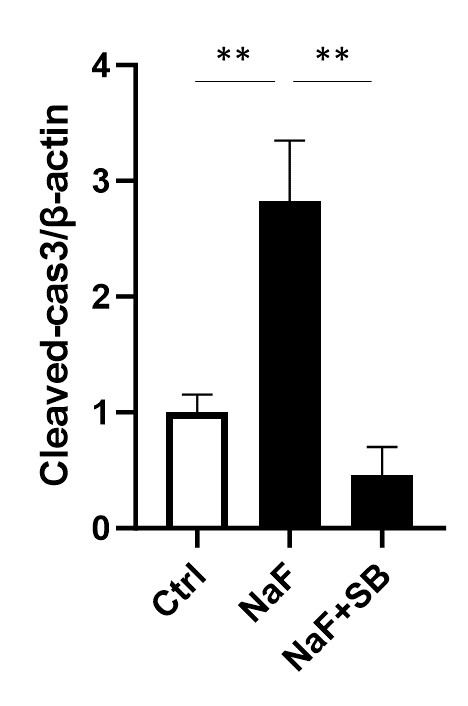

Supplement: Supplementary file 1 [file ijms-25-09600-s001.zip › Fig S5.jpg]

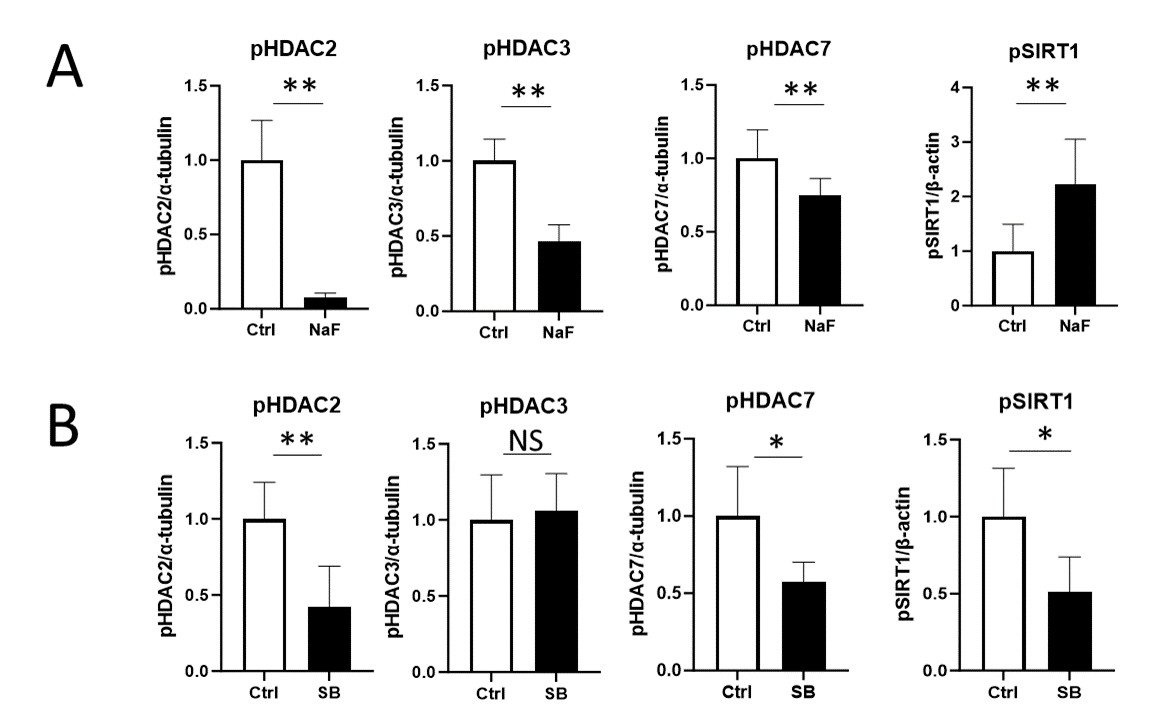

Supplement: Supplementary file 1 [file ijms-25-09600-s001.zip › FigS1.jpg]

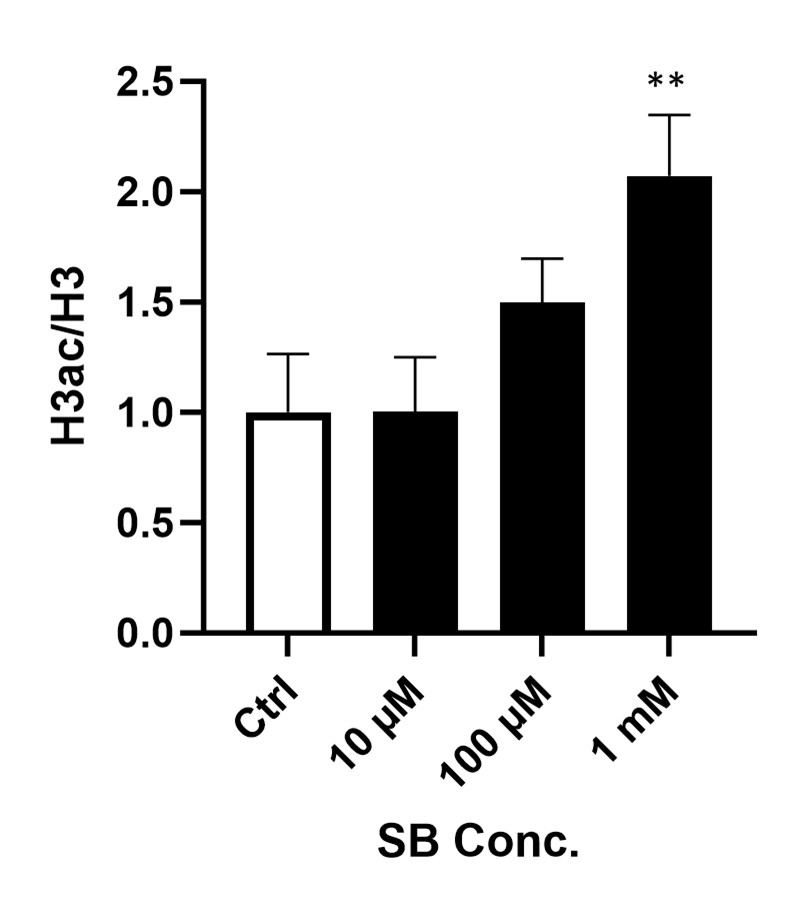

Supplement: Supplementary file 1 [file ijms-25-09600-s001.zip › FigS2.jpg]

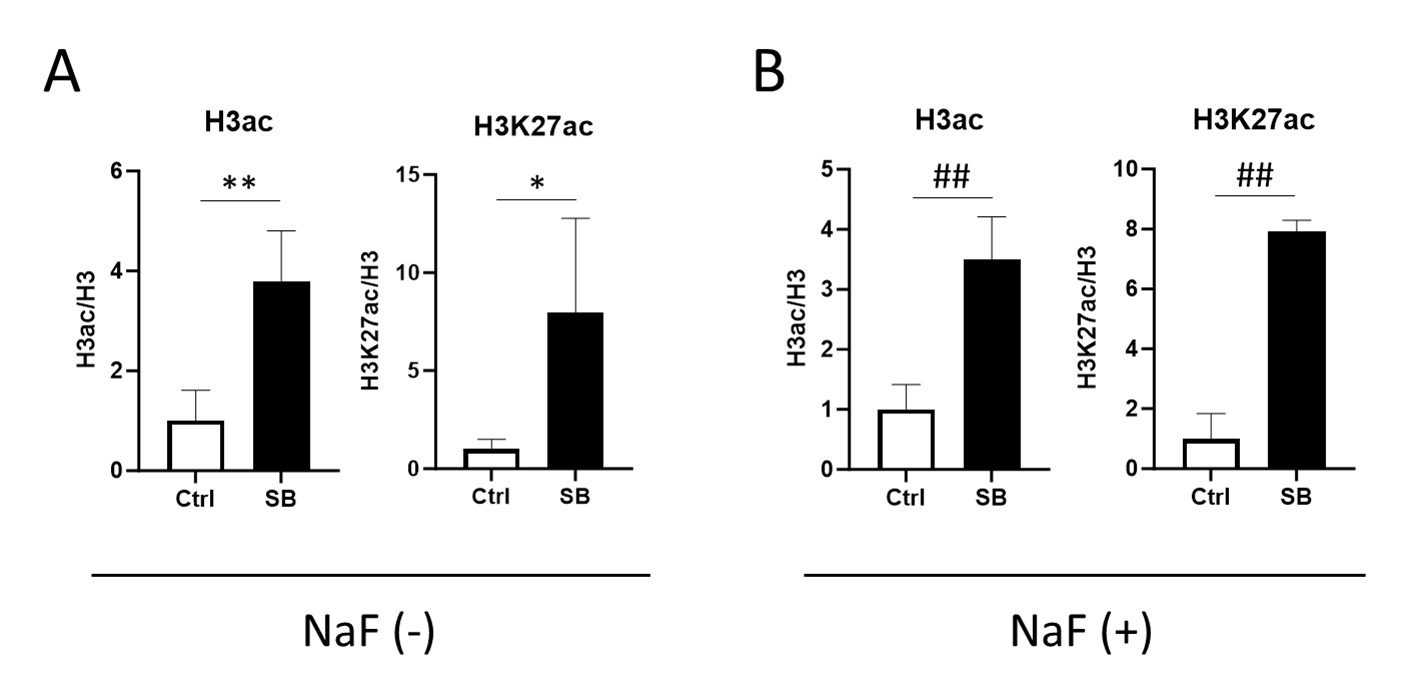

Supplement: Supplementary file 1 [file ijms-25-09600-s001.zip › FigS3.jpg]

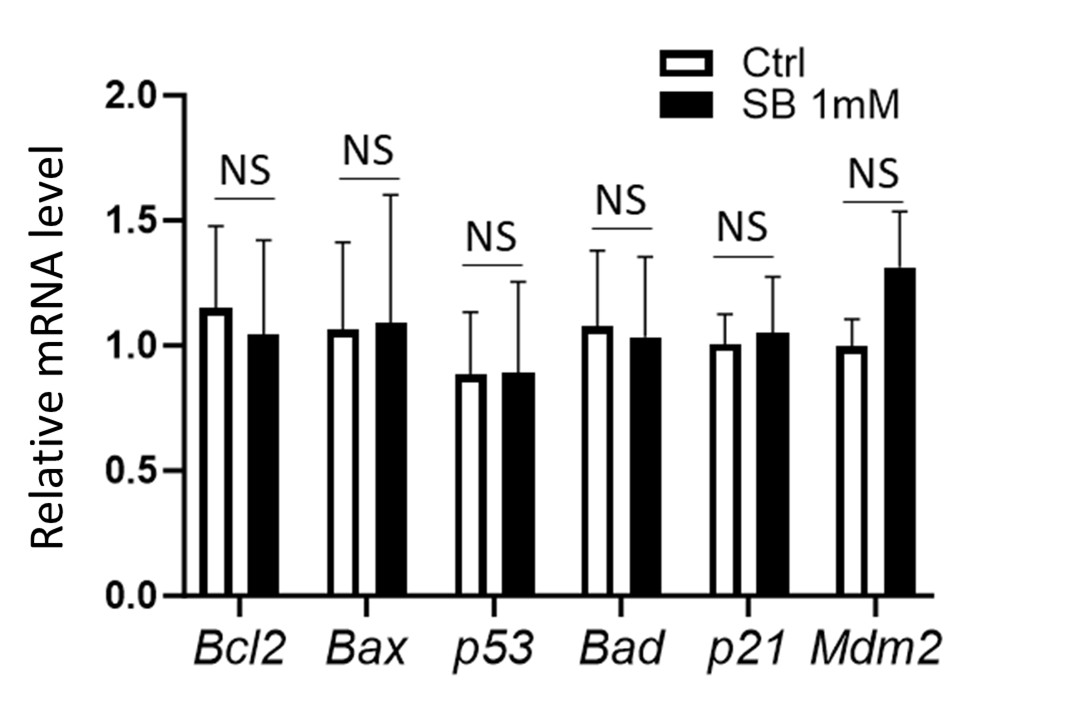

Supplement: Supplementary file 1 [file ijms-25-09600-s001.zip › FigS4.jpg]

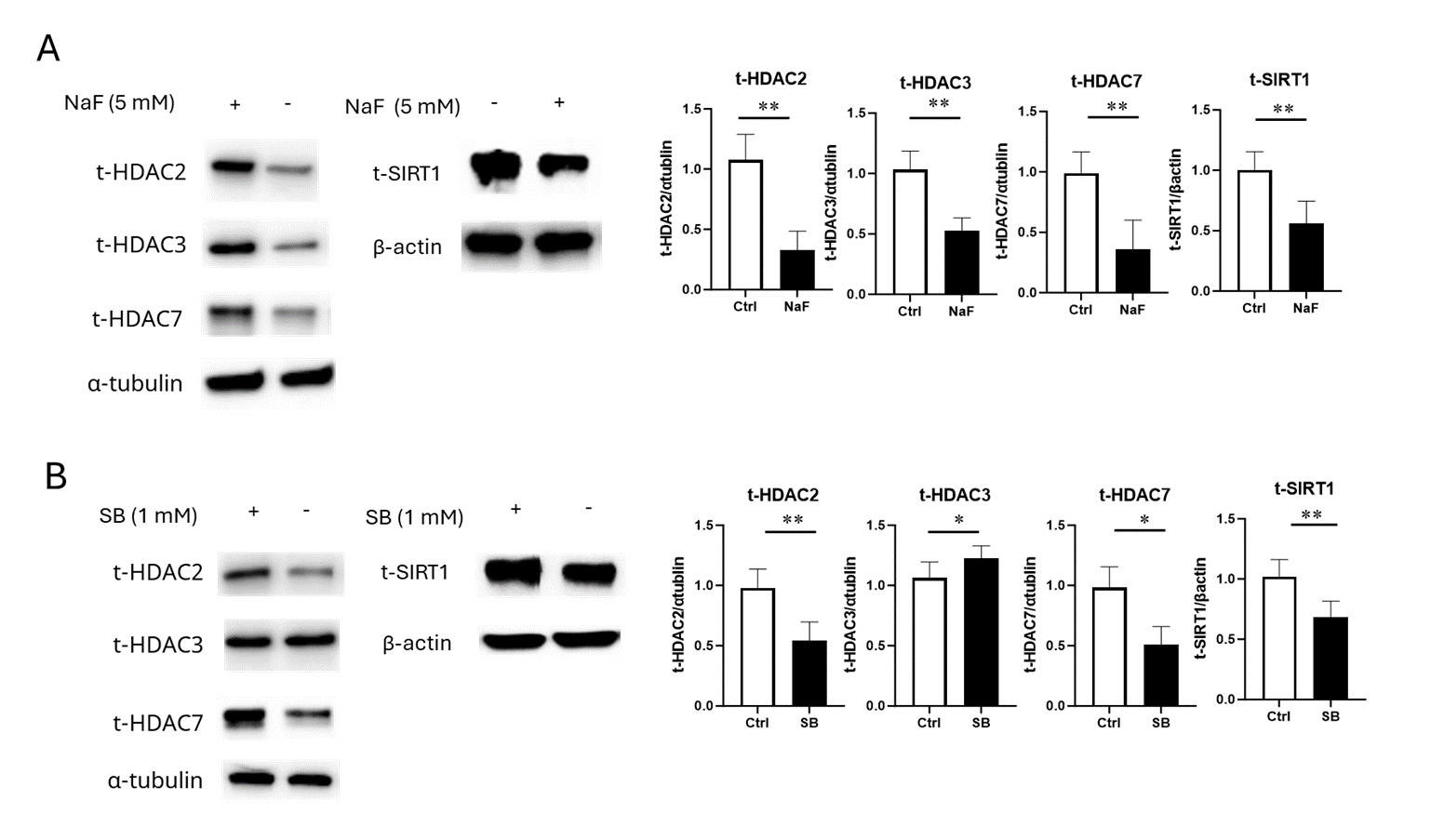

Supplement: Supplementary file 1 [file ijms-25-09600-s001.zip › Supp S6.jpg]

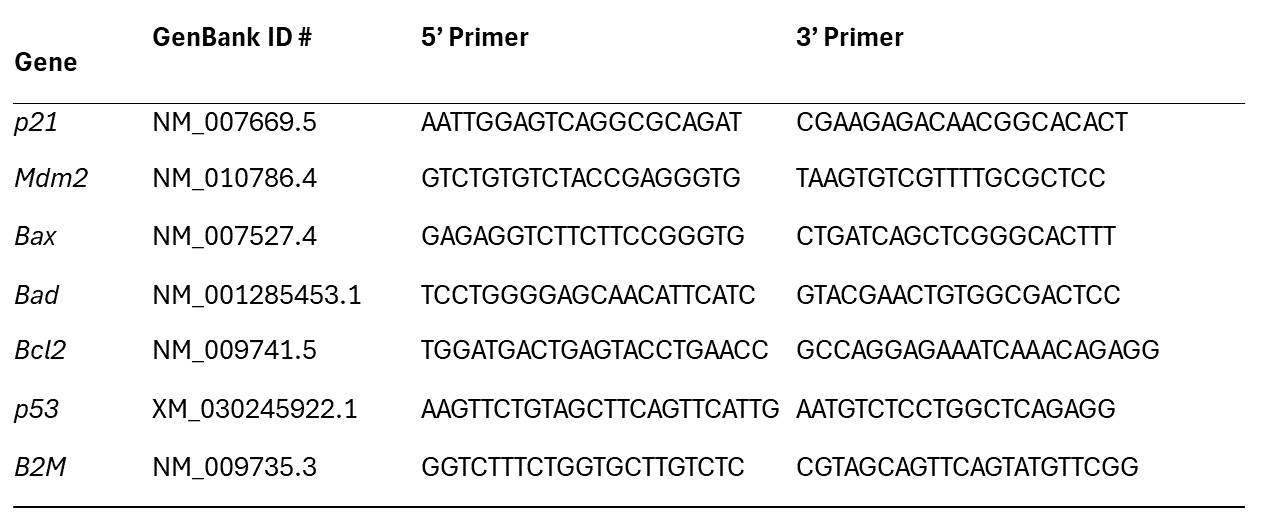

Supplement: Supplementary file 1 [file ijms-25-09600-s001.zip › Table S1.jpg]
